# New insights on atherosclerosis: A cross-talk between endocannabinoid systems with gut microbiota

**DOI:** 10.15171/jcvtr.2018.21

**Published:** 2018-09-27

**Authors:** Jalal Moludi, Mohammad Alizadeh, Ned Lotfi Yagin, Yahiya Pasdar, Seyed Mostafa Nachvak, Hadi Abdollahzad, Ali Sadeghpour Tabaei

**Affiliations:** ^1^Nutrition Research Center, Faculty of Nutrition, Tabriz University of Medical Sciences, Tabriz, Iran; ^2^Students’ Research Committee, Tabriz University of Medical Sciences, Tabriz, Iran; ^3^Nutritional Sciences Department, School of Nutritional Sciences and Food Technology, Kermanshah University of Medical Sciences, Kermanshah, Iran; ^4^Rajaie Cardiovascular Medical and Research Center, Iran University of Medical Sciences, Tehran

**Keywords:** Atherosclerosis, Endocannabinoids, Gastrointestinal Microbiome, Probiotics, Trimethylamine-N-Oxide

## Abstract

The incidence of atherosclerosis is increasing rapidly all over the world. Inflammatory processes have outstanding role in coronary artery disease (CAD) etiology and other atherosclerosis manifestations. Recently attentions have been increased about gut microbiota in many fields of medicine especially in inflammatory diseases like atherosclerosis. Ineffectiveness in gut barrier functions and subsequent metabolic endotoxemia (caused by rise in plasma lipopolysaccharide levels) is associated with low-grade chronic inflammation i.e. a recognized feature of atherosclerosis. Furthermore, the role of trimethylamine-N-oxide (TMAO), a gut bacterial metabolite has been suggested in atherosclerosis development. On the other hand, the effectiveness of gut microbiota modulation that results in TMAO reduction has been investigated. Moreover, considerable evidence supports a role for the endocannabinoid system (ECS) in atherosclerosis pathology which affects gut microbiota, but their effects on atherosclerosis are controversial. Therefore, we presented some evidence about the relationship between gut microbiota and ECS in atherosclerosis. We also presented evidences that gut microbiota modulation by pre/probiotics can have significant influence on the ECS.

## Introduction

Cardiovascular disease (CVD) is one of the most common causes of mortality throughout the world with high social costs in terms of health care.^1^ Atherosclerosis, the main cause of CVD development, which is considered as an inﬂammatory disease and created by arterial lesions containing cholesterol, immune inﬁltrates, and connective tissue elements.^2^ In addition to the known risk factors such as hyperlipidemia and smoking which might raise endothelial injury, inﬂammatory processes may be involved in the development of atherosclerosis manifestations.^3^ Recently attentions have been increased about gut microbiota in many fields of medicine especially in inflammatory diseases like atherosclerosis.^4^ Furthermore, gut microbiota imbalance may be a key player in the inflammation onset in other organs which could alter the regular homeostasis.^5-7^ However, the intestinal and molecular components involved in gut-to-organ dysregulation in atherosclerosis still remain unknown.^8^ Moreover, our current understanding of the pathophysiology of atherosclerosis suggests that endocannabinoid signaling plays a critical role in pathogenesis of the atherosclerosis. The potential role of endocannabinoid in atherosclerosis seems to be their modulation of the chronic inﬂammatory response that occurs within the vascular wall.^9^

In the current review, we debate recent findings that reveal mechanisms connecting the gut microbiota to low-grade inflammation in context of the obesity. Additionally, we will discuss the potential relationships between the endocannabinoid systems (ECSs) with gut microbiota. Lastly, we talk over the potential modulation and their effects on gut microbiota and host metabolism in regard to development of the atherosclerosis.

## Gut Microbiota

The human large intestine is consisted of large and various communities of microbial cells that create the gut microbiota. The whole microbial genome of the gut microbiota is known as the gut microbiome, which is 100 times greater than the human genome indicating the importance of gut microbiome.^8-10^ There is no clear definition of healthy gut microbiota in human, but in healthy individuals, anaerobic *Bacteroidetes* and *Firmicutes* constituted more than 90% of the total bacterial species.^11,12^

Bacterial diversity among individuals is due to the differences in both host genomes and environmental factors, such as antibiotic usage, lifestyle, hygiene, and diet.^13-15^ An altered gut microbial composition, known as dysbiosis, may lead to unfavorable effects in host and could predispose an individual to disease.^16^

Although, the increased microbial diversity contributes to greater stability of the gut environment and better health status, the mechanism is unknown.^17-19^ Previous studies have shown that the gut microbiota can also affect a number of complex metabolic reactions in other organs possibly through gut permeability, fat storage and low-grade inflammation,^20,21^ which subsequently affect atherogenesis. According to present knowledge, immune responses have a prominent role in atherosclerosis pathophysiology.^22,23^ Considering the gut microbiota effects in immune systems,change in gut microbiota would have a valuable influence on atherosclerosis.^20^ It seems that gut microbiota affects the atherosclerosis process in three ways. First, the chronic inflammation induced by metabolic endotoxemia, second by harmful metabolites such as trimethylamine-N-oxide (TMAO) due to the gut microbial dysbiosis and also because of the potential relationships between the ECSs with gut microbiota.

## 1) Systemic inflammation induced by dysbiosis

**Figure 1:**
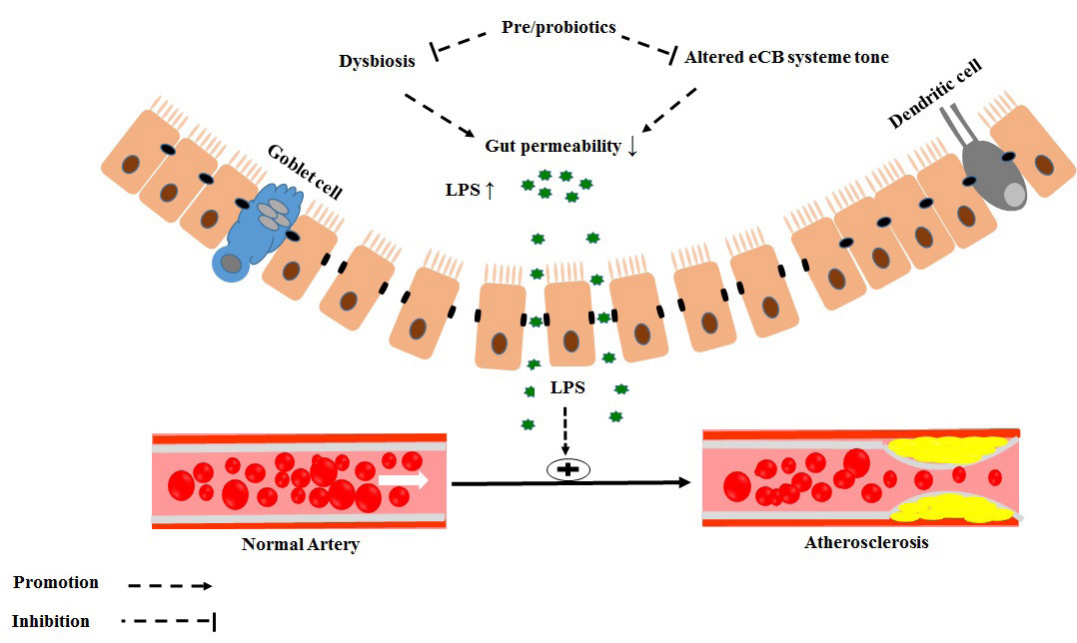
Endocannabinoid systems affect a number of complex metabolic reactions which might affect gut microbiota. According to current understanding, changes in gut microbiota have prominent role in the pathophysiology of atherosclerosis. Alter in ECS system tone increases gut permeability which enhances the LPS level. In fact there are two ways for increasing the LPS: 1) alter the tone of gut microbiota and 2) ECS system.

Several recent studies have proposed that mucosal barrier function disruption known as “dysbiosis” and subsequent gut microbiota-derived endotoxemia could lead to cardiometabolic diseases pathogenesis.^24,25^ Increased levels of plasma endotoxin through chylomicrons absorption have been associated with CVD development.^26^ Animal studies also revealed the accelerated atherosclerosis after endotoxins injection.^27^ In this process, bacterial translocation and endotoxins are recognized by Toll-like receptors (TLRs) expressed on the host macrophages which in turn results in chronic inflammation. TLRs are family of membrane pattern-recognition receptors, which have a vital role in immune system and protect against many pathogens. It should be also mentioned that stimulation with lipopolysaccharides (LPS) recognized by TLRs (Figure 1) can lead to uncontrollable proinflammatory cytokines production, which can result in CVD.^28,29^ In addition, this permits LPS leakage into the circulation activating systemic inflammation cytokines like tumor necrosis factor alpha (TNF-α) and interleukin 6 (IL-6) production.^30^ Immunological and inflammatory processes probably play an important role in atherosclerosis pathogenesis and progression. Pro-inflammatory cytokines, such as IL-1, TNF-α, C-reactive protein (CRP), IL-2, and IL-6, all increase in patients with atherosclerosis.^31^

Some studies have demonstrated that probiotics can decrease the pro-inflammatory cytokines production.^32,33^ Though in clinical trials, antibiotic therapy and subsequent gut microbiota alteration did not improve secondary cardiovascular outcomes,^34,35^ it seems that, microflora intervention by diet modulation had better effects than antibiotic therapy. In both human and animal studies, probiotic supplementation was associated with reduced cholesterol profile and inhibition of atherosclerotic lesion composition and development.^36^ For example, 8 weeks *Saccharomyces boulardii* supplementation in hypercholesterolemic adults led to decrease in lipoprotein particles and cardiovascular biomarkers.^37^ It has been accepted that probiotics decrease inflammation and endotoxemia. However, the precious mechanism underlying remains unclear. But it is proposed that intestinal barrier plays an important role through lessening the translocation of microbiome-derived LPS into the bloodstream.

## Obesity and dysbiosis

Obesity was already known as low-grade inflammation and some studies confirmed that obesity is linked with changes in gut microbiota in comparison with lean counterparts.^38,39^ In fact, it can be assumed that the increased inflammation is the main reason of the CVD in obese individuals.^40^ On the other hand, due to the impaired gut barrier function and altered gut microbiota, an obese person is more susceptible to inflammation.^41^ For example, Cani et al, have shown that in obese mice, intestinal permeability and metabolic endotoxemia play major part in metabolic disorders pathogenesis. Moreover, they found that changes in gut microbiota through prebiotic supplementation increased endogenous glucagon like peptide (GLP-2 ) production, which in turn improved gut barrier functions, decreased inflammatory and oxidative stress markers.^5^

## Gut microbiota modulation

To date, therapeutic methods which have been used to modify the microbiota include antibiotics, diet, prebiotics/probiotics.^34,42-44^ While antibiotics therapy can alter the intestinal microflora, this method has failed to improve cardiovascular outcomes in clinical trial. Furthermore, considering the possible antibiotic resistance and microbial changes, this method has not been effective yet.^34,45^ Prebiotics are non-digestible food elements that stimulate the growth activity of the gut bacteria.^22^ Probiotics, the live microorganisms, have positive effects the host by producing vitamin K, B2, and short chain fatty acids such as acetate or propionate which are used as fuels for intestinal flora and coloncytes. Additionally, they decrease the epithelial barriers permeability in intestine, increase tight junctions’ protein function, interact with host microbial community and compete with pathogens. Probiotics strains also interact with toll-like receptors in gut modifying the inflammatory responses.^43,46-48^ A recent systematic review and meta-analysis of randomized controlled trials showed that probiotic supplementation did not only improve lipid profile and cardiovascular risk, but supplements with multiple probiotic strains was more useful rather than a single strain,^47^ suggesting the importance of intestinal microflora diversity.

As mentioned above, probiotics have beneficial effects on cardiovascular system. In this regard, a recent study revealed that *Lactobacillus rhamnosus,* a probiotic supplement decreased cardiac hypertrophy and improved ejection fraction at an animal model with myocardial infarction.^49^ Therefore, regarding all these together the importance of probiotics effects in inflammation reduction as a main reason of atherosclerosis can be deduced.

## 2) TMAO as a marker of cardiovascular events

Although, bacterial components cause inflammation, certain bacterial metabolites such as trimethylamine^50^ can also exert cytotoxicity and increase inflammation. Trimethylamine a biological compound is produced by gut microbiota from dietary phosphatidylcholine, choline, and carnitine. Liver flavin monooxygenase (FMO) enzymes oxidize TMA TMAO releasing it into the circulation.^50,51^ TMAO concentrations are 3 µmol/L and 40 µmol/L in blood of healthy subjects and in renal failure patients respectively.^52^ It should also be considered that TMAO levels may be affected by many factors including amount of dietary choline, carnitine, gut microbiota activity and kidney function.^53^ Therefore, due to the considerable influence of TMAO in CVD, these factors especially kidney function should also be considered. In previous epidemiological studies, being vegetarian was associated with lower risk of CVD.^54-56^ On the other hand, high content of TMAO precursors in omnivores diet with different intestine micro-flora (compared to vegetarians, omnivores had more *Prevotella* and lower *Bacteriodes* species in gut microbiome) led to higher susceptibility of these individuals to atherosclerosis.^57,58^ It seems that TMAO might contribute to atherosclerosis development in part by activating macrophages to accumulate cholesterol to form foam cells in atherosclerotic lesions. Also, TMAO could change cholesterol metabolism in different organs such as liver and intestines inhibiting reverse cholesterol transport pathway.^59^ As well, elevated TMAO levels have been shown to have prognostic value in patients with ischemic and non-ischemic cardiomyopathy.^60^ Recent studies have assessed the effects of intestinal environment manipulation in reducing TMAO levels.^61,62^ Wang et al demonstrated that in addition to decreased foam cell formation, TMAO production was prevented by 3, 3-dimethyl-1-butanol (DMB).^61^ In another study Rong et al^62^ presented that resveratrol supplementation inhibited the TMAO production by changing intestinal microflora, and supplementation also changed gut microbes type and increased Lactobacillus and Bifidobacterium species levels. In an animal study, probiotic usage changed TMAO levels which subsequently reversed atherosclerosis development.^63^ Gut microbiota modulation in some patients may be the alteration of TMAO levels.^64-66^ Wang et al^56^ showed that gut microbiota plays a significant role in TMAO production in animal models. They indicated that treating mice with antibiotics changed the intestinal microflora and subsequently decreased TMAO production. Also, in a study conducted by Tripolt et al, daily supplementation of *Lactobacillus casei* Shirota (6.5 × 10^9^ CFU) 3 times for 12 weeks in subjects with metabolic syndrome did not affect TMAO levels.^53^ Consequently, it seems that well-designed clinical trials with different probiotic sources are required to determine the impact of probiotics on TMAO production.

Effect of nutritional intervention on TMAO levels is inconsistent. For example, one study has found that while fish consumption was beneficial for CVD, the TMAO levels also increased concurrently.^52^ In another study, oral L-carnitine supplementation significantly decreased vascular injury markers such as intercellular adhesion molecule-1 (ICAM), (vascular cellular adhesion molecule-1)VCAM-1 and malondialdehyde (MDA); however, at the same time plasma TMAO level increased significantly.^67^ Information in this area is limited and need more research in order to find the best modulation in this direction. Moreover, a previous study has shown that oxidative stress plays an important role in cardiovascular and neurodegenerative diseases such as Alzheimer’s disease and Parkinson’s disease.^68^ Recent studies have highlighted TMAO positive role in CVD by reducing oxidative stress.^69,70^ In a study, TMAO administration for about 12 weeks decreased endoplasmic reticulum stress and peripheral nerves dysfunction in streptozotocin-diabetic rats’ models.^69^ However, it is noteworthy to mention that it has not proved that TMAO promotes atherosclerosis and different food sources are more likely to be useful and confounding factors should be considered in the interpretation in this field.

## 3) Endocannabinoid systems

The ECS is an internal signaling system acts in various physiological functions, both in central and peripheral nervous systems and also in peripheral organs.^71^ 2-arachidonyl glycerol (2-AG) and anandamide are main endocannabinoids which bind to cannabinoid receptors, named cannabinoid receptor 1 (CB 1) and CB 2, that are G-protein-coupled membrane receptors with an identical signaling mechanism (rReviewed elsewhere).^52^ CB1 was first found in distinct areas of the brain as well as peripheral nerve terminals. CB2 is located in lymphoid tissues and myeloid cells and have role in immune response.^67^ However, the expression of these receptors has not been restricted to such areas. For example, the CB2 receptor is also expressed in myocardium, coronary artery, endothelial and smooth muscle cells in cardiovascular system. Additionally, CB1 receptors have also been identiﬁed in myocardium, human coronary endothelial and smooth muscle cells.^50,70^

The mechanisms by which endocannabinoids act as cardio protective components, include decreased neutrophil inﬁltration, inﬂammation, oxidative stress and increased activation of cardio protective signaling pathways through activation of CB1 and CB2.^68^ Modulation of immunity and inflammatory response which occurs within the vascular wall appears to be the main role of endocannabinoid in atherosclerosis prevention.^69^ The anti-inﬂammatory effects of CB2 activation are improvement of endothelial function, proliferation of vascular smooth muscle cell, plaque development, expression of adhesion molecules, decrease in oxidative stress and macrophage inﬁltration in in-vivo system.^70,72^ Though, CB2 deﬁciency increased atherosclerosis susceptibility in mice,^73^ due to the evidence of both pro- and anti-atherosclerotic effect of receptor activation, the role of CB1 in atherosclerosis is still controversial.^74,75^ AEA and 2-AG release from endothelial due to the endocannabinoids administration had inconsistent effects ranging from increased heart rate and blood pressure to reduced atherosclerosis progression in mice.^76,77^ In addition, low dose of anandamide lessened tumor necrosis factor a (TNFa) levels and induced ICAM-1, VCAM-1 expression in human coronary artery endothelial cell.^78^ In contrast to theoretically beneficial effects of endocannabinoids in CVD, they might also demonstrate pro-thrombotic effects. As well, in previous animal and human studies it was observed that AEA and 2-AG activated platelet aggregation leading to atherosclerosis progression.^79,80^ Considering the endocannabinoid side effects, it seems that their modulation with different method such as probiotics supplements might minimize their negative effects.^59^ In addition to modulation of the host’s immune responses and decline of harmful metabolite such as TMAO by probiotics, they might also stimulate CB1 and CB2 receptors.

## Obesity and endocannabinoid system

As stated above, overweight and obesity are associated with increased gut permeability leading to metabolic endotoxemia and CVD. Also, obesity is associated with over activation of the ECS and gut microbiota modulation with prebiotics leads to normalization of ECS tone. On the other hand, gut-derived LPS contributes to altered endocannabinoid tone. Hence, the greater ECS tone which is found in obesity might also participate in CVD not only directly by acting on cardiovascular risk factors, but also indirectly by increasing plasma LPS levels, that consequently impair the gut permeability.

## Interaction between Gut modulation and endocannabinoid systems

As discussed above, atherosclerosis is associated with altered ECS system tone.^81^ Muccioli et al showed that gut microbiota selectively controls ECS system tone and also demonstrated that LPS is a potent stimulator of ECS synthesis. They also revealed that ECS system could regulate gut permeability and adipogenesis and gut microbiota modulation by probiotics strongly affects this pathway.^82^ Dietary supplementation with probiotics decreased gut permeability, increased the expression of CB2 mRNA and lessened CB1 mRNA expression, AEA production and fat mass storage (Table 1). In fact, it can be said that gut microbiota and ECSs affect each other. In addition to direct role of ECS in cardiovascular system, it also plays a vital role in regulating gut-barrier function. The concept of gut-barrier is expressed by two concepts ‘gate keepers’ or ‘gate openers’^83^ and probiotics generally mediate the gate keepers’ role.^84^ In fact, these functions in gut permeability control are not only related, they are identical. Rousseaux et al also showed a link between gut microbiota and ECS. They demonstrated that oral *Lactobacillus acidophilus* strain administration modulated cannabinoid receptors expression in rats’ intestinal cells expression.^85^ Furthermore, tight junction proteins (Zonulin and occluding) expression in Caco-2 cells were controlled by CB1-dependent mechanisms, but not by CB2.^82,86^ Changes in CB1 and CB2 expression were associated with amount of bacterial translocation. Previous study showed that CB2 receptor activation, improved glucose tolerance and reduced permeability in an animal model study.^87^

**Table 1:** Effects of gut modulation on EC system

**Subjects**	**Intervention**	**Effect**	**Reference**
Mice	Feeding *L. acidophilus*	Increased expression of CB2 mRNA in colonic epithelial cells Analgesic functions in the gut comparable with the effects of endocannabinoid	Rousseaux et al^85^
*Solea solea*	Probiotic treatment with *Enterococcus faecium*	Up-regulated CB_1_ mRNA Modulate energy homeostasis by inducing endocannabinoid signaling	Palermo et al^88^
Ob/ob mice	Prebiotic treatment with oligofructose	Lessen CB1 mRNA expression, AEA production and fat mass storage in prebiotics group LPS levels correlated with CB_1_ mRNA expression and AEA levels	Muccioli et al^82^
Mice	Microbiota manipulations with antibiotics	2.5-fold decrease in total bacterial counts levels Up-regulation of CB2 expression	Aguilera et al^88^

One of the primary disadvantages of non-selective stimulation of CB receptor by endocannabinoid administration is its psychotropic side effects. However, pro/prebiotics administration seems to be well-tolerated without any harm. A recent study has shown that changing intestinal microflora with probiotics modified CB2 receptor expression. In this study, it was revealed that microbiota manipulations with antibiotics was associated with 2.5-fold decrease in the level of total bacterial counts and CB2 expression up-regulation in mice.^89^ In another study it was also demonstrated that *Lactobacillus acidophilus* administration increased CB2 expression in mice colon, whereas some specific bacteria strain such as *Bifidobacterium* and* Escherichia coli* had no effect on CB2 expression.^85^ Briefly, it seems that probiotics have undeniable role in ECS stimulation; however, according to the literature review, this area needs further investigation to exactly determine which bacteria strain has more beneficial effects depending on the different receptor. Further clinical studies are required to elucidate the cross-talk between probiotic of ECS system

## Interaction between atherosclerosis, endocannabinoid systems and gut microbiota

Gut microbiota also regulates gut permeability and inflammatory response through LPS-ECS system regulatory loops.^82,90^ On the other hand, LPS stimulates ECS synthesis,^77,91^ hyper-activates it in the intestines leading to increase in gut permeability and systemic inflammation.^90^ The crosstalk between gut microbiota and ECS system might contribute to atheroscrosis development directly by acting on adipose tissue and indirectly by increasing plasma LPS levels.^82^ Probiotic may also alter gut microbiota tone which in turn decreases both intestinal and adipose tissue ECS system responsiveness, improving gut barrier and stabilize adipogenesis. It has been shown that blocking CB_1_ receptor protects against low-grade inflammation which is promising evidence that the ECS system, inflammation, and atherosclerosis are interrelated.^91^ Therefore, we suggest that there might be similar molecular mechanisms underlying the ECS and gut barrier function related to endotoxemia and inflammation.

## Conclusion

Even though there are many questions which have been unanswered, studies demonstrated that mucosal barrier function disruption and subsequent gut microbiota-derived endotoxemia could contribute to cardiometabolic diseases pathogenesis. As well, number of studies revealed that TMAO in systemic circulation can activate macrophages which lead to cholesterol accumulation and subsequent foam cells formation in atherosclerotic lesions. On the other hand, accumulating evidence proposes that ECS involved in many physiological processes that are related to maintenance of gut-barrier function and inflammation regulation. Hence, although present literature review provides beneficial evidence in support of crosstalk between ECS and gut microbiota, additional studies are needed to clarify whether gut microbiota modulation can alter ECS tone and inflammation levels or not.

## Ethical approval

Not applicable.

## Competing interests

All authors declare no competing financial interests exist.

## Acknowledgments

The authors would like to thanks, Rajaie Heart Hospital doctors who helped us.

## Key points

- Gut microbiota interact with host via several mechanisms such as control of intestinal permeability, prevention of endotoxin (LPS) absorption and subsequent inflammation
- ECS involved in many biological processes, ranging from the regulation of energy homeostasis to inflammation and intestinal barrier function
- Gut microbiota regulates gut permeability and inflammatory response through LPS-eCB system regulatory loops
- Gut microbiota modulation by pre/probiotics can have significant influence on the ECS
